# Analytical performance evaluation of MAGLUMI HBeAg and anti-HBe chemiluminescence immunoassays and comparison with the Abbott assays

**DOI:** 10.3389/fcimb.2026.1867239

**Published:** 2026-08-19

**Authors:** Jingfeng Zeng, Xin Zhao, Wenfeng Wang, Ju Wang, Ximiao Li, Kaize Zheng, Hongwei Zhang, Jun Yin, Zhonggang Fang

**Affiliations:** 1 Blood Screening Department, Shenzhen Blood Center, Shenzhen, China; 2 Medical Research and Laboratory Diagnostic Center, Jinan Central Hospital Affiliated to Shandong First Medical University, Jinan, China; 3 Research & Development Department, Shenzhen New Industries Biomedical Engineering Co., Ltd. (Snibe), Shenzhen, China

**Keywords:** analytical performance, chemiluminescence immunoassay, hepatitis B e antibody, hepatitis B e antigen, method comparison

## Abstract

**Background:**

Hepatitis B e antigen (HBeAg) and its corresponding antibody (anti-HBe) serve as critical serological markers in the evaluation and therapeutic management of Hepatitis B virus (HBV) infection.

**Study design:**

This retrospective study was designed to evaluate the performance of the newly CE-marked MAGLUMI HBeAg (CLIA) and MAGLUMI Anti-HBe (CLIA) assays by comparing with Abbott Architect HBeAg and Abbott Architect Anti-HBe assays, respectively. Analytical performance was verified in compliance with the guidelines issued by the Clinical and Laboratory Standards Institute (CLSI). Negative and positive specimens were collected to determine negative percent agreement (NPA) and positive percent agreement (PPA) respectively. Analytical specificity was assessed using negative samples containing potentially cross-reacting substances. Additionally, analytical sensitivity was evaluated using 1st WHO International Standard.

**Results:**

The claimed performance specifications for LoB, LoD, linearity, and precision were successfully verified. Both MAGLUMI HBeAg (CLIA) and MAGLUMI Anti-HBe (CLIA) assays exhibited satisfactory NPA and PPA (≥98%). No significant cross-reactivity or interference was observed. Furthermore, all tested plasma matrices demonstrated equivalence to serum in both assays.

**Conclusions:**

The MAGLUMI HBeAg (CLIA) and MAGLUMI Anti-HBe (CLIA) met the CLIS verification criteria with favorable analytical performance, and achieved high NPA and PPA relative to the Abbott Architect comparative assays. Besides, future concordance assessments based on a large intended cohort, together with independent clinical validation, are required to fully confirm their suitability for routine clinical application and management of HBV infection.

## 1 Introduction

Hepatitis B virus (HBV), a small DNA virus with retrovirus-like features, is a major global cause of liver diseases and hepatocellular cancer (Liang, 2009; Iannacone and Guidotti, 2022). Globally, HBV infection accounts for approximately 30% of cirrhosis and 53% of liver cancer. The virus has infected roughly one-third of the global population, resulting in nearly 360 million chronic carriers. Besides, among chronic carriers, about one million deaths occur annually due to HBV-related liver disease (Hwang and Cheung, 2011; Neuveut et al., 2010). Despite the World Health Organization’s goal to eliminate viral hepatitis by 2030, the elimination of HBV remains challenging due to its efficient transmission routes (vertical and horizontal transmission) and its ability to establish persistent infection within host cells (Hwang and Cheung, 2011; Stevens et al., 1975; Chang, 2007).

Infection with wild-type HBV leads to the appearance of characteristic serological markers in blood. These include the hepatitis B surface antigen (HBsAg) and its antibody (anti-HBs), anti-HBc antibodies (IgM/IgG), and the hepatitis B e antigen (HBeAg) and its antibody (anti-HBe) (de Almeida Pondé, 2021; Watson et al., 2025). HBeAg, a secretory protein derived from the precore region, shares substantial sequence homology with the core protein (HBcAg). It is regarded as an HBV accessory protein as it is not required for viral infection, replication, or assembly (Chen et al., 2017). HBeAg plays a crucial role in establishing persistent infection *in vivo* by modulating host immunity. Evidence indicates that the secreted HBeAg induces T-cell tolerance far more effectively than HBcAg (Chen et al., 2005; Ferrari, 2015). Anti-HBe is the antibody produced by the human immune system in response to the HBeAg. The clinical interpretation of anti-HBe relies on the context of other HBV markers, especially HBeAg. Clinically, serological markers of HBV are commonly used to assess different stages of HBV infection and guide the management of patients. The presence of HBeAg specifically indicates active HBV replication and high transmissibility in both acute and chronic infection (Ferrari, 2015; Kao, 2008). Importantly, it should be noted that HBeAg detection is highly time-dependent during the initial acute phase of HBV infection. False-negative results may occur if sampling is performed either too early or too late relative to the time of viral exposure. The presence of anti-HBe signifies early immune recovery and, at least theoretically, reduces the risk of progression to chronic infection. Furthermore, this seroconversion event predicts the subsequent emergence of neutralizing anti-HBs antibodies, thereby indicating a resolved infection and immune recovery (de Almeida Pondé, 2021).

Although the HBeAg/anti-HBe profile does not provide definitive criteria for classifying the clinical form of infection, it offers valuable insights for patient evaluation (de Almeida Pondé, 2021). Moreover, the assessment of the HBeAg/anti-HBe profile should be interpreted in conjunction with other HBV serological markers for comprehensive clinical evaluation. Annual global mortality from Hepatitis B virus (HBV) and its complications is expected to increase by 39% from 2015 to 2030 due to widespread under-diagnosis and under-treatment (Hsu et al., 2023). Besides, compared to the traditional enzyme-linked immunosorbent assay (ELISA), the chemiluminescence immunoassay (CLIA) exhibits superior sensitivity, short turnaround time, and improved consistency and reproducibility in the detection of serum markers (Li et al., 2023; Shen et al., 2024; Wang et al., 2024; Fang et al., 2025).

To address the need for better HBV management, the MAGLUMI HBeAg and MAGLUMI Anti-HBe chemiluminescence immunoassays (CLIAs) have been developed for the evaluation and therapeutic management of HBV infection. This study was designed to verify their analytical performance following CLSI protocol, and further evaluate the assay agreement by comparison with well-established commercial assays.

## 2 Materials and methods

### 2.1 MAGLUMI HBeAg assay

**Figure 1:**
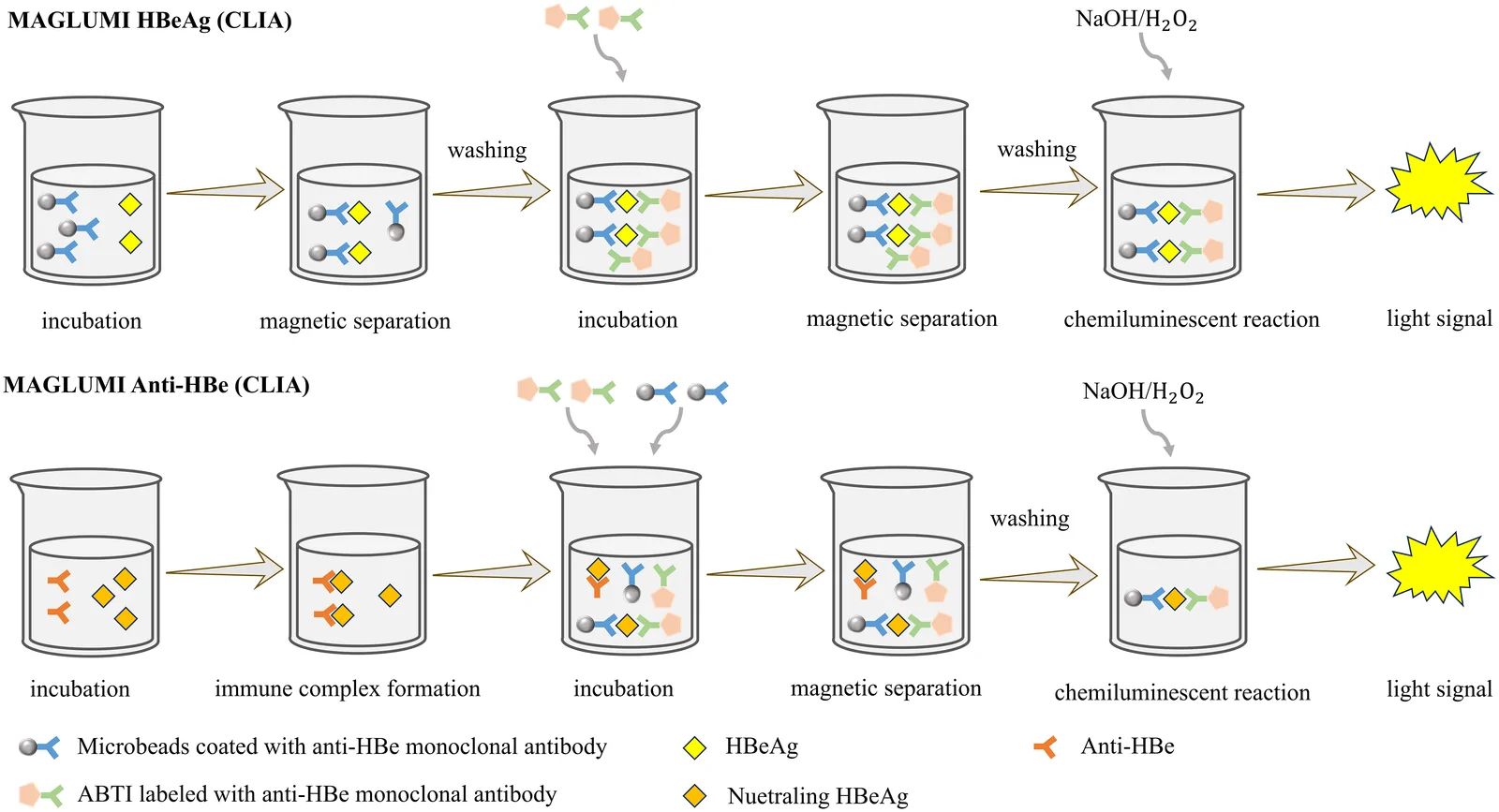
Schematic illustration of the MAGLUMI HBeAg (CLIA) and Anti-HBe (CLIA) test principles.

The MAGLUMI HBeAg (CLIA) assay is a sandwich chemiluminescence immunoassay. Sandwich complexes are formed among the sample, anti-HBe monoclonal antibody solid phase, and another anti-HBe monoclonal antibody labeled with N-(4-Aminobutyl)-N-ethylisoluminol (ABEI). After magnetic separation and washing, the chemiluminescent reaction is initiated by adding NaOH and H_2_O_2_. The resulting light signal is proportional to the concentration of HBeAg in the sample (**Figure 1**). A result greater than the cut-off value (0.1 IU/mL) is considered positive. The precision and accuracy of the assay were monitored using Snibe Hepatitis Controls (HQC).

### 2.2 MAGLUMI anti-HBe assay

The MAGLUMI Anti-HBe (CLIA) assay is a competitive chemiluminescence immunoassay. Nuetralizing HBeAg is combined with anti-HBe in the sample to form an immune complex firstly. Sandwich complexes are formed among the neutralizing HBeAg, anti-HBe monoclonal antibody solid phase, and another anti-HBe monoclonal antibody labeled with N-(4-Aminobutyl)-N-ethylisoluminol (ABEI). After magnetic separation and washing, the chemiluminescent reaction is initiated by adding NaOH and H_2_O_2_. The resulting light signal is proportional to the concentration of anti-HBe present in the sample (**Figure 1**). A result greater than the cut-off value (1.0 AU/mL) is considered positive. The precision and accuracy of the assay were monitored using Acctribu Unassayed Complex Infectious Diseases Controls (IDQC(U)).

### 2.3 Verification of analytical performance

#### 2.3.1 Detection capability of HBeAg assay

The verification of the limit of blank (LoB) and the limit of detection (LoD) was performed according to the CLSI EP17-A2 guideline. For LoB verification, two blank sample matrices were tested in replicates of n=4 on each of three consecutive days. For LoD verification, separate aliquots of the same blank matrix were spiked to a concentration of 0.100 IU/mL and analyzed using the same replication scheme.

#### 2.3.2 Linearity of HBeAg assay

The proportional linearity of MAGLUMI HBeAg (CLIA) assay was analyzed over the entire assay range following the guideline of CLSI EP06-A2. Spiked samples with low and high concentrations were mixed in ratios of 8:2, 6:4, 4:6 and 2:8 to obtain 6 distinct concentration levels. Each sample was tested in duplicate.

#### 2.3.3 Precision of HBeAg assay

Three samples with distinct concentrations were analyzed over five independent runs each day for 5 testing days. The within-run and between-day precision were assessed following the CLSI EP05-A3 guideline.

#### 2.3.4 Interference of HBeAg and anti-HBe assays

Potential endogenous and exogenous interferents were added to three samples with different analyte concentrations, as described in CLSI EP07-A2. The interference effect was calculated based on the difference in analyte concentration before and after the addition.

### 2.4 Method comparison

The method comparison of the MAGLUMI HBeAg (CLIA) and MAGLUMI Anti-HBe (CLIA) assays was evaluated in accordance with the Common Technical Specifications (Decision 2002/364/EC and amendments). The comparative methods were the CE-marked Abbott Architect HBeAg (REF 6C32) and Anti-HBe (REF 6C34) assays, with a cut-off of 1.0 S/CO. For HBeAg, results “≥” the cut-off are positive; for Anti-HBe, results “≤” the cut-off are positive.

#### 2.4.1 Study population

**Figure 2:**
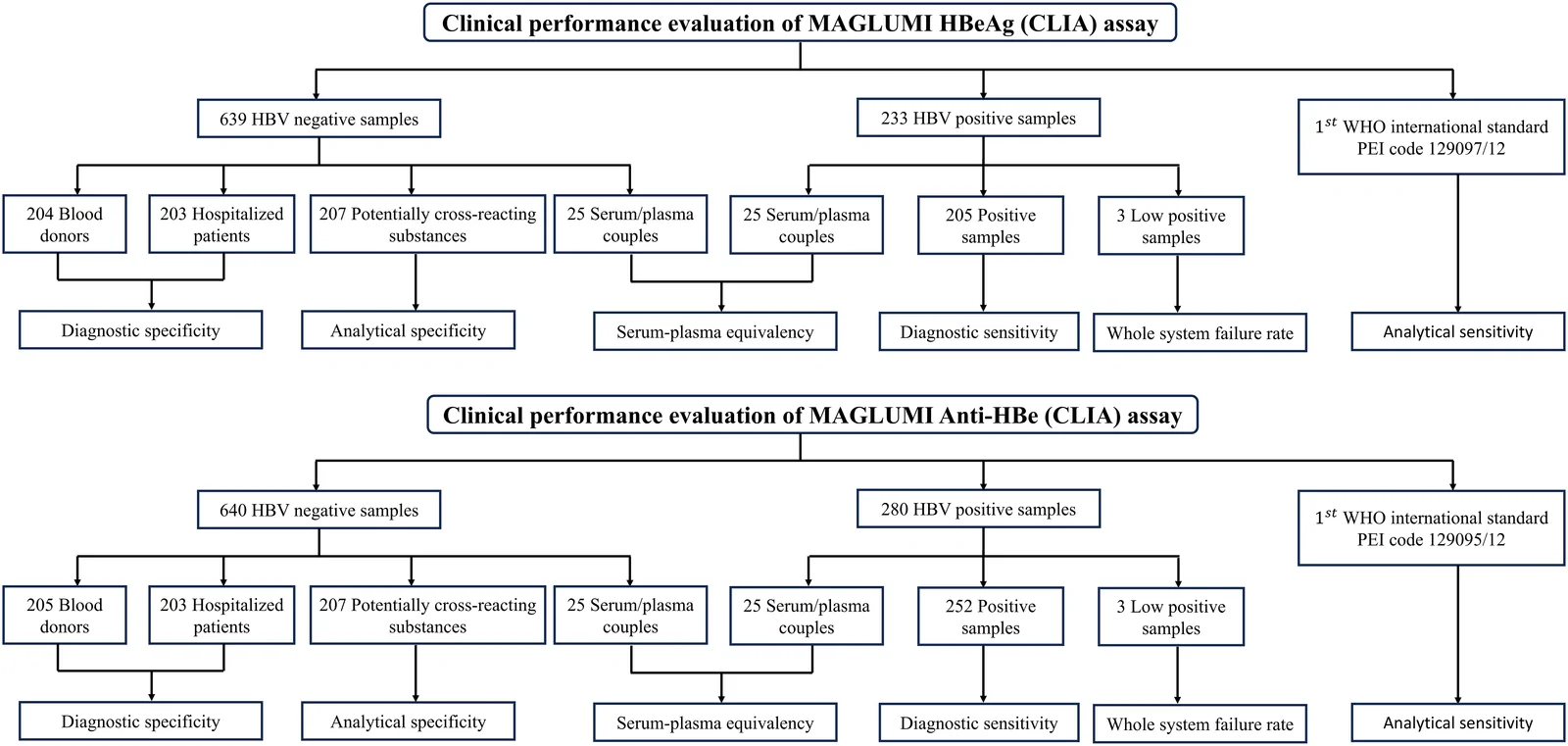
Study flow diagram.

Blood donors were enrolled consecutively, while the other clinical samples were selected by random sampling (**Figure 2**).

#### 2.4.2 Analytical specificity

207 potentially cross-reacting samples (**Figure 2**) expected to be non-infected with HBV were tested with the MAGLUMI HBeAg (CLIA), MAGLUMI Anti-HBe (CLIA), Architect HBeAg and Architect Anti-HBe assays to determine the analytical specificity.

#### 2.4.3 Analytical sensitivity

Three independent two-fold dilution series (7 dilutions, 0.016–1.000 IU/mL) of the 1^st^ WHO HBeAg International standard (PEI code 129097/12)(**Figure 2**) and the 1^st^ WHO anti-HBe International standard (PEI code 129095/12) were prepared and tested with MAGLUMI HBeAg (CLIA) and MAGLUMI Anti-HBe (CLIA) assays, respectively. Analytical sensitivity was defined as the assigned concentration of each standard corresponding to the respective assay cut-off value.

#### 2.4.4 Negative percent agreement

204 HBV negative blood donations, 203 clinical samples of hospitalized patients (**Figure 2**) expected to be non-infected with HBV were tested with the MAGLUMI HBeAg (CLIA) and Architect HBeAg assays to determine the NPA. 205 HBV negative blood donations, 203 clinical samples of hospitalized patients expected to be non-infected with HBV were tested with the MAGLUMI Anti-HBe (CLIA) and Architect Anti-HBe assays to determine the NPA.

#### 2.4.5 Positive percent agreement

205 HBeAg positive samples (**Figure 2**) were tested with the MAGLUMI HBeAg (CLIA) and Architect HBeAg assays to determine the PPA. 252 anti-HBe positive samples were tested with the MAGLUMI Anti-HBe (CLIA) and Architect Anti-HBe assay to determine the PPA.

#### 2.4.6 Serum to plasma equivalency

25 negative and 25 spiked positive serum and plasma (Sodium-Citrate, K2EDTA, K3EDTA, Lithium Heparin, CPD, CPDA, Sodium Heparin, ACD-B, K-Oxalate/Soduim-Fluoride) (**Figure 2**) pairs were tested using the MAGLUMI HBeAg (CLIA) and MAGLUMI Anti-HBe (CLIA) assays. Serum and plasma were considered equivalent if the same interpretation was obtained.

#### 2.4.7 Whole system failure rate

Whole system failure rate was determined as the number of false negative results on three low-positive samples (**Figure 2**) for 22 days.

### 2.5 Statistical analysis

Excel (2019) and R software (Version 4.4.1) were employed to analyze the mean values, standard deviations, coefficient of variation, two-sided 95% confidence intervals, and regression equations. *p* “<“ 0.05 is considered statistically significant. Passing-Bablok analysis was used only for serum- plasma equivalency, and was not adopted for the method comparison of the MAGLUMI and Abbott assays.

### 2.6 Ethics

The method comparison was conducted by an independent, third-party laboratory (Biomex GmbH, Heidelberg, Germany) and the analytical performance verification study was conducted by the Blood Screening Department, Shenzhen Blood Center in accordance with the ethical principles of the Declaration of Helsinki. This study use residual samples covered under a general ethical approval protocol (Ethics No.:SZBCMEC-2025-056), with broad consent provided by all subjects. All residual samples were collected and tested in Germany from May 18, 2021 to June 24, 2021 and in China from March 20, 2025 to June 22, 2025.

## 3 Results

### 3.1 Analytical performance

The claimed LoB and LoD for MAGLUMI HBeAg (CLIA) assay are 0.010 IU/mL and 0.100 IU/mL, respectively. In this verification, only one of 24 results (0.026 IU/mL) exceeded the claimed LoB, which met the minimum acceptance criterion of 87% for a sample size of 24 at 95% confidence level as specified in CLSI EP17-A2. Similarly, all 24 results were at or above the claimed LoD, confirming the LoD claim. Additionally, as shown in **Supplementary Figure 1**, a strong linear relationship was observed from 0.010 to 177.000 IU/mL (R^2^ = 0.9902). Furthermore, the within-run CV ranged from 1.24% to 2.54%, and the between-day CV from 0.00% to 1.59% (**Table 1**), demonstrating excellent precision of the MAGLUMI HBeAg (CLIA) assay. The acceptance limit for potential interferents was within ±10%, and no tested interferents exhibited interference in either the HBeAg or Anti-HBe assays (**Supplementary Table 1**).

**Table 1:** Precision of MAGLUMI HBeAg (CLIA) assay.

Assay	Sample	Mean (IU/mL)	Within-run		Between-day		Total	
			SD	% CV	SD	% CV	SD	% CV
MAGLUMI HBeAg (CLIA)	S1	0.511	0.013	2.540	0.000	0.000	0.013	2.540
	S2	50.036	0.848	1.690	0.000	0.000	0.848	1.690
	S3	152.520	1.897	1.240	2.427	1.590	3.081	2.020

### 3.2 Analytical specificity

A total of 207 negative cross-reactive samples were tested to evaluated analytical specificity. For the MAGLUMI HBeAg (CLIA) assay, only one sample from an influenza-positive individual was positive (**Table 2**). This result was interpreted as a false positive because it was found negative on both the Architect HBeAg assay and confirmatory Roche Cobas HBeAg assay. For the Anti-HBe assay, all samples from the same cohort were identified as negative, which demonstrates its reliability to distinguish anti-HBe from potential interfering substances.

**Table 2:** Analytical specificity of MAGLUMI HBeAg (CLIA) and MAGLUMI anti-HBe (CLIA) assays.

Cross-reacting/ interfering samples	MAGLUMI HBeAg (CLIA)		Architect HBeAg		MAGLUMI anti-HBe(CLIA)		Architect anti-HBe	
	Negative	Positive	Negative	Positive	Negative	Positive	Negative	Positive
HAMA (autoimmume)	5	0	5	0	5	0	5	0
Rheumatoid Factor	27	0	27	0	27	0	27	0
Hyper IgG/IgM	13	0	13	0	13	0	13	0
HAV	8	0	8	0	8	0	8	0
HCV	22	0	22	0	22	0	22	0
HEV	14	0	14	0	14	0	14	0
CMV IgM	8	0	8	0	8	0	8	0
EBV IgM	14	0	14	0	14	0	14	0
VZV IgG	4	0	4	0	4	0	4	0
HSV 1/2 IgG	15	0	15	0	15	0	15	0
Influenza	20	1	21	0	21	0	21	0
HIV Ag/Ab	7	0	7	0	7	0	7	0
Syphilis	12	0	12	0	12	0	12	0
Pregnant women	22	0	22	0	22	0	22	0
Dialysis patients	15	0	15	0	15	0	15	0
Total	206	1	207	0	207	0	207	0
Analytical specificity	99.52% (95% CI [97.34% - 99.99%])		100.00% (95% CI [98.23% - 100.00%])		100.00% (95% CI [98.23% - 100.00%])		100.00% (95% CI [98.23% - 100.00%])	

HAV, hepatitis A virus; HCV, hepatitis C virus; HEV, hepatitis E virus; CMV, cytomegalovirus; EBV, Epstein-Barr virus; VZV, varicella-zoster virus; HSV, herpes simplex virus; HIV, human immunodeficiency virus.

### 3.3 Analytical sensitivity

The analytical sensitivity was determined using the designated standard materials of 1^st^ WHO International standard PEI code 129097/12 and PEI code 129095/12 (Wang et al., 2021; Commission, 2022). In this study, two-fold dilution series of the 1^st^ WHO International standard were assayed in triplicate. The assigned concentration of the HBeAg standard corresponding to the 0.1 IU/mL cut-off value of the MAGLUMI HBeAg (CLIA) assay was 0.084 IU/mL, while the assigned concentration of the anti-HBe standard corresponding to the 1.0 AU/mL cut-off value of the MAGLUMI Anti-HBe (CLIA) assay was 0.219 IU/mL (**Supplementary Table 2**).

### 3.4 Negative percent agreement

All 204 negative blood donor samples and 203 negative hospitalized samples were found negative with both the MAGLUMI HBeAg (CLIA) and Architect HBeAg assays (**Table 3**), corresponding to a NPA of 100% (*n* = 407, 95%CI: 99.10%–100.00%) (**Table 4**). For the MAGLUMI Anti-HBe assay, all 205 blood donor samples were tested negative, whereas 2 of 203 negative hospitalized samples were found positive. These two positive samples were confirmed as false positive via the third-party Roche Cobas Anti-HBe assay (**Table 5**, **Supplementary Table 3**). The NPA of MAGLUMI Anti-HBe was therefore calculated as 99.51% (*n* = 408, 95% CI: 98.23%–99.94%).

**Table 3:** 2×2 table reporting cross-classification of results generated by the MAGLUMI HBeAg (CLIA) and architect HBeAg assays.

MAGLUMI HBeAg	Architect HBeAg		
	Positive	Negative	Total
Positive	205	0	205
Negative	0	407	407
Total	205	407	612

**Table 4:** Assay agreement of MAGLUMI HBeAg (CLIA) and MAGLUMI anti-HBe (CLIA) assays.

Assay method	Measure	Calculation	Value	95% CI
MAGLUMI HBeAg assay	NPA	407/407	100.00%	99.10%-100.00%
	PPA	205/205	100.00%	98.16%-100.00%
	PPV	205/205	100.00%	98.16%-100.00%
	NPV	407/407	100.00%	99.10%-100.00%
	OPA	612/612	100.00%	99.38%-100.00%
	Kappa coefficient	/	1.0000	1.0000-1.0000
MAGLUMI Anti-HBe assay	NPA	406/408	99.51%	98.23%-99.94%
	PPA	247/252	98.02%	95.44%-99.35%
	PPV	247/249	99.20%	97.12%-99.90%
	NPV	406/411	98.78%	97.18%-99.60%
	OPA	653/660	98.94%	97.83%-99.49%
	Kappa coefficient	/	0.9775	0.9609-0.9941

NPA, negative percent agreement; PPA, positive percent agreement; PPV, positive predictive value; NPV, negative predictive value; OPA, Overall Percent Agreement.

**Table 5:** 2×2 table reporting cross-classification of results generated by the MAGLUMI anti-HBe (CLIA) and Architect anti-HBe assays.

MAGLUMI anti-HBe	Architect anti-HBe		
	Positive	Negative	Total
Positive	247	2	249
Negative	5	406	411
Total	252	408	660

### 3.5 Positive percent agreement

The MAGLUMI HBeAg (CLIA) assay exhibited 100% PPA (95% CI: 98.16%–100.00%) in 205 positive samples, among which 15 were from acute infection patients and 15 from chronic infection patients. For MAGLUMI Anti-HBe (CLIA) assay, 5 negative results were obtained from 252 anti-HBe positive samples. These five samples were confirmed false negative via the third-party Roche Cobas Anti-HBe assay (**Supplementary Table 4**). Thus, the PPA of the MAGLUMI Anti-HBe (CLIA) assay was determined to be 98.02% (*n* = 252, 95%CI: 95.44%–99.35%).

### 3.6 Serum to plasma equivalency

25 negative and 25 positive serum-plasma pairs were tested using the MAGLUMI HBeAg (CLIA) and MAGLIMI Anti-HBe (CLIA) assays. All negative pairs exhibited negative results in both assays (**Supplementary Table 5**). All sample from the positive pairs were positive in both assays. However, three discrepant results were observed in MAGLUMI HBeAg (CLIA) assay. Since these three results were significantly lower compared to the others in the same sample group, they are classified as outliers. To quantitatively evaluate serum-plasma equivalency for the MAGLUMI HBeAg (CLIA) assay, Passing-Bablok regression analysis was performed after excluding outliers.

#### 3.6.1 Passing-Bablok regression analysis for MAGLUMI HBeAg assay with positive sample pairs

To evaluate whether differences existed between serum and the different plasma matrices, the data were further analyzed according to CLSI EP09-A3. The estimated correlation coefficients and the results of the Passing-Bablok regression (estimates and 95% CI) are present in **Supplementary Table 6** and **Supplementary Figure 2**. The regression coefficients demonstrated excellent agreement between all plasma types and serum, as all slopes approached 1 and all intercepts approached 0.

### 3.7 Whole system failure rate

The 3 low positive HBeAg samples and 3 low positive anti-HBe samples were tested over 22 different days within a 5-week period. All tests were interpreted as positive (**Supplementary Table 7**). The calculated whole system failure rate for both MAGLUMI HBeAg (CLIA) and MAGLUMI Anti-HBe (CLIA) assays was 0% (0/120).

## 4 Discussion

The claimed LoB, LoD and linearity were successfully verified for MAGLUMI HBeAg (CLIA). As shown in **Table 1**, the within-run precision (%CV) for low, medium, and high concentration samples (2.540%, 1.690%, 1.240%, respectively) was comparable to that of the Abbott Architect HBeAg assay (Wang et al., 2017). Furthermore, common endogenous interferents (hemoglobin, lipids, and bilirubin) and Drugs frequently used in hepatitis or co-infection therapy (e.g., theophylline, entecavir, ampicillin, telbivudine, and metronidazole) were evaluated. None of these substances exhibited significant interference for both MAGLUMI HBeAg (CLIA) and MAGLUMI Anti-HBe (CLIA).

Analytical specificity refers to an assay’s ability to exclusively distinguish and measure the intended analyte without interference from other substances in the sample (Rambla-Alegre et al., 2012). Cross-reactivity is a significant concern in diagnostic immunoassays. It can occur through three mechanisms: (1) the presence of endogenous molecules structurally similar to the target analyte; (2) analyte metabolites sharing common cross-reacting epitopes; or (3) the administration of structurally similar medications to the patient (Kroll and Elin, 1994). Cross-reacting influenza antibodies were induced *in vivo* after influenza virus infection (Chiu et al., 2013), potentially leading to false-positive result in the MAGLUMI HBeAg (CLIA) immunoassays.

The analytical sensitivity, also known as detection limit, is defined as the lowest amount of an analyte in a sample that can be quantified accurately by an assay (Saah and Hoover, 1997). Although no detailed acceptance criteria were dictated in Common Technical Specifications (CTS) 2002/364/EC and amendments, the analytical sensitivity of MAGLUMI HBeAg (CLIA) assay was comparable to that of Elecsys-HBeAg and Architect-HBeAg assays (Wang et al., 2021). Moreover, the MAGLUMI HBeAg (CLIA) assay exhibited a lower limit of analytical sensitivity (0.084 IU/mL) compared with the Vitros Immunodiagnostic HBeAg assay (129 IU/mL), the ETI-EBK PLUS HBeAg assay (97 IU/mL), and even the FDA-approved ADVIA Centaur HBeAg assay (1 IU/mL) (Mixson-Hayden et al., 2018). Notably, the assigned concentrations cannot be compared directly, as they are calibrated against separate WHO standards corresponding to the cut-off value of MAGLUMI HBeAg (CLIA) assay and MAGLUMI Anti-HBe (CLIA), respectively.

A method comparison study was conducted between the investigational MAGLUMI HBeAg (CLIA) assay and the Architect HBeAg assay. The Architect HBeAg assay served as the comparative method due to its proven reliability and well-established performance characteristics (Maylin et al., 2013; Song et al., 2025; Choi et al., 2013). The MAGLUMI HBeAg (CLIA) assay demonstrated 100% NPA (n=407), indicating that positive results are highly likely to be true positives. Besides, the MAGLUMI Anti-HBe (CLIA) assay yielded two false-positive results, corresponding to a NPA of 99.51% (n=408). No lipemia, hemolysis or icterus was observed in the two false-positive samples. Theoretically, there are several reasons for false-positive results in CLIA assays, such as autoimmune rheumatoid factor (RF), heterophile antibodies, or other interfering substances (Tate and Ward, 2004). Specifically, heterophile antibodies can bridge the solid-phase capture antibodies and labeled antibodies, generating a false signal chain and a false-positive result (Levinson and Miller, 2002; Bouvet et al., 2001). Given that the two false-positive samples in this study originated from hospitalized patients, heterophile antibodies are considered a potential explanation for the observed false-positive results. Besides, prolonged specimen storage and repeated freeze-thaw cycles can lead to degradation of serum proteins, which might generate fragments that trigger non-specific binding.

In this study, the MAGLUMI HBeAg (CLIA) assay and MAGLUMI Anti-HBe (CLIA) assay demonstrated PPA of 100% and 98.02%, respectively. The endogeneous native HBeAg in patient samples may compete with neutralizing HBeAg for anti-HBe binding, which could leave excess unreacted neutralizing antigen and potentially yield false-negative results. Besides, these five discordant results are likely induced by matrix effects. Overall, the NPA and PPA of both MAGLUMI HBeAg (CLIA) and MAGLUMI Anti-HBe (CLIA) assays not only meet the ≥98% acceptance criteria of the Common Technical Specifications (CTS) 2002/364/EC and amendments, but also showed higher PPA and NPA than Abbott Alinity assays and Fujirebio Lumipulse assays in comparative studies against the Abbott Architect assays (Choi et al., 2013; Kutvonen et al., 2022).

Serum-plasma equivalency, defined as the consistency of results obtained from serum and plasma matrices, is a critical performance characteristic for a CLIA assay (Li et al., 2023). Both the MAGLUMI HBeAg (CLIA) and MAGLUMI Anti-HBe (CLIA) assays showed excellent agreement for negative serum-plasma pairs. For positive serum-plasma pairs, three outliers may be attributed to locally elevated anticoagulant concentrations resulting from inadequate mixing. However, the quantitative nature of MAGLUMI HBeAg (CLIA) assay and three outliers necessitated a formal statistical evaluation for serum-plasma equivalency. Analysis of the quantitative data revealed that the magnitude of the differences was proportional to the analyte concentration, indicating a constant %CV over the range. Therefore, Passing-Bablok regression was employed and it confirmed a strong correlation for positive serum-plasma pairs. Furthermore, the inclusion or exclusion of outliers results in nearly identical slope and intercept values and does not change clinical interpretation (**Supplementary Table 6**).

The whole system failure rate is the frequency of failures when the entire process is performed as prescribed by the manufacturer. In this study, the whole system failure rate was reported as the number of false negative results on the total number of repeated assays on the low-positive samples (Commission, 2022). Both MAGLUMI HBeAg (CLIA) and MAGLUMI Anti-HBe (CLIA) assays exhibited a whole system failure rate of 0%, confirming robust performance and result reliability.

A key limitation of this study is the absence of a gold standard comparative method. Quantitative PCR (qPCR) is widely regarded as the gold standard for HBV diagnosis and monitoring due to its high sensitivity and broad range for viral load quantification (Navvabi et al., 2022). Without validation against a gold standard, great agreement between investigational and comparative assays does not conclusively confirm the reliability of the investigational assay. Moreover, limited enrollment of relevant HBV subgroups and blood donors is another major limitation. Our positive cohort only included patient with acute, chronic and recovered infection. Inactive carriers, treated patients, individuals undergoing HBeAg seroconversion, patients coinfected with HIV, HCV, or tuberculosis, and those with complications such as liver fibrosis, cirrhosis, or hepatocellular carcinoma were absent. In addition, blood donor specimens derive from low-risk populations with minimal endogenous interferents such as heterophilic antibodies and rheumatoid factors, which introduces selection bias and tends to overestimate NPA. Therefore, larger-scale concordance evaluations with representative cohorts and additional independent clinical validations will be necessary to thoroughly establish the assays’ reliability for the routine application and management of HBV infection. Moreover, an HBeAg-positive/anti-HBe-negative serological profile signifies a high risk of transmission, whereas an HBeAg-negative/anti-HBe-negative profile signifies a low risk. There are 4 different transition phases (Immune-tolerant phase, HBeAg positive immune-active phase, HBeAg negative immune-active phase, and inactive carrier phase) in chronic HBV infection (de Almeida Pondé, 2021). Future studies should include chronic infection samples to evaluate the utility of HBeAg/anti-HBe profile in classifying the phase of chronic HBV infection. However, the MAGLUMI Anti-HBe (CLIA) assay is only qualitative, while there is a growing demand and trend for quantitative assays (Caviglia et al., 2019; Numan et al., 2025). It is reported that an anti-HBe titer > 0.68 IU/ml served as a specific predictor (sensitivity = 44.4% and specificity = 95.9%) for intrahepatic HBV cccDNA (Caviglia et al., 2019). Therefore, a quantitative anti-HBe assay should be developed to guide treatment decisions and assess residual risks after clinical cure (i.e., HBsAg negative).

In terms of clinical significance, although numerous commercial assays are available for detecting these two markers, most of them require large-scale matched analytical instruments. The assay-compatible instruments MAGLUMI X3 and MAGLUMI X8 match distinct laboratory requirements. The high-throughput MAGLUMI X8 is designed for large-scale central laboratories with heavy daily tests, while the compact MAGLUMI X3 system fits well with resource-limited community medical facilities, enabling multi-level coverage of clinical testing scenarios.

In conclusion, the primary finding of this work is the strong assay agreement observed between the MAGLUMI HBeAg/Anti-HBe CLIAs and the Abbott Architect assays. Secondly, this study comprehensively characterizes the analytical performance of these newly CE-marked MAGLUMI assays in strict accordance with CLSI guidelines and enables broad coverage across various clinical laboratory settings.

## Data Availability

The original contributions presented in the study are included in the article/**Supplementary Material**. Further inquiries can be directed to the corresponding authors.
