# Supplementary material for: Analytical performance evaluation of MAGLUMI HBeAg and anti-HBe chemiluminescence immunoassays and comparison with the Abbott assays
Source: Front Cell Infect Microbiol. 2026 Aug 19;16:1867239. doi: 10.3389/fcimb.2026.1867239 (PMC13533807; doi:10.3389/fcimb.2026.1867239)
Supplement: Supplementary file 1 [file Table1.docx]

Supplementary Material

Supplementary Table S1 Interference evaluation of MAGLUMI HBeAg (CLIA) and MAGLUMI Anti-HBe (CLIA) assays

| Interferent | Interferent concentration  (mg/dL) | MAGLUMI HBeAg (CLIA) | | MAGLUMI Anti-HBe (CLIA) | |
| --- | --- | --- | --- | --- | --- |
|  |  | Analyte  (IU/mL) | Interference | Analyte  (AU/mL) | Interference |
| Hemoglobin | 250 | 0.575 | 8.90% | 0.267 | 0.00% |
|  | 250 | 4.92 | 2.50% | 1.656 | 1.04% |
|  | 250 | 26.2 | 3.97% | 10.556 | 2.69% |
|  | 500 | 0.605 | 4.36% | 0.267 | 0.00% |
|  | 500 | 4.94 | 2.92% | 1.661 | 1.34% |
|  | 500 | 25.8 | 2.38% | 10.223 | -0.54% |
| Lipid | 1000 | 0.541 | 5.46% | 0.267 | 0.00% |
|  | 1000 | 4.55 | -2.57% | 1.567 | -4.39% |
|  | 1000 | 24.3 | -2.80% | 10.279 | 0.00% |
|  | 2000 | 0.484 | -5.65% | 0.267 | 0.00% |
|  | 2000 | 4.3 | -7.92% | 1.65 | 0.67% |
|  | 2000 | 23.1 | -7.60% | 9.39 | -8.65% |
| Bilirubin | 10 | 0.551 | 1.10% | 0.267 | 0.00% |
|  | 10 | 4.87 | 1.04% | 1.606 | -2.01% |
|  | 10 | 25.3 | 1.20% | 10.39 | 1.08% |
|  | 20 | 0.534 | -2.02% | 0.267 | 0.00% |
|  | 20 | 4.76 | -1.24% | 1.667 | 1.71% |
|  | 20 | 25.3 | 1.20% | 10.501 | 2.16% |
| Theophylline | 5 | 0.544 | 2.06% | 0.267 | 0.00% |
|  | 5 | 4.87 | 0.62% | 1.489 | -9.15% |
|  | 5 | 25.7 | 0.39% | 9.89 | -3.78% |
|  | 10 | 0.553 | 3.75% | 0.267 | 0.00% |
|  | 10 | 4.73 | -2.27% | 1.545 | -5.74% |
|  | 10 | 24.6 | -3.91% | 9.945 | -3.25% |
| Entecavir | 0.25 | 0.538 | -1.82% | 0.267 | 0.00% |
|  | 0.25 | 4.7 | 0.86% | 1.6 | -2.38% |
|  | 0.25 | 25.1 | 1.21% | 10.223 | -0.54% |
|  | 0.5 | 0.524 | -4.38% | 0.267 | 0.00% |
|  | 0.5 | 4.75 | 1.93% | 1.545 | -5.74% |
|  | 0.5 | 24.5 | -1.21% | 9.945 | -3.25% |
| ampicillin | 50 | 0.532 | -1.30% | 0.267 | 0.00% |
|  | 50 | 4.52 | -1.53% | 1.739 | 6.10% |
|  | 50 | 24.1 | 1.26% | 10.445 | 1.61% |
|  | 100 | 0.539 | 0.00% | 0.267 | 0.00% |
|  | 100 | 4.35 | -5.23% | 1.617 | -1.34% |
|  | 100 | 23.9 | 0.42% | 11.279 | 9.73% |
| Telbivudine | 30 | 0.532 | 0.00% | 0.267 | 0.00% |
|  | 30 | 4.73 | -1.87% | 1.55 | -5.43% |
|  | 30 | 23.8 | -0.83% | 9.945 | -3.25% |
|  | 60 | 0.533 | 0.19% | 0.267 | 0.00% |
|  | 60 | 4.67 | -3.11% | 1.483 | -9.52% |
|  | 60 | 24.3 | 1.25% | 10.723 | 4.32% |
| metronidazole | 10 | 0.513 | -0.77% | 0.267 | 0.00% |
|  | 10 | 4.52 | -3.62% | 1.533 | -6.47% |
|  | 10 | 25.1 | 2.87% | 9.89 | -3.78% |
|  | 20 | 0.51 | -1.35% | 0.267 | 0.00% |
|  | 20 | 4.7 | 0.21% | 1.556 | -5.06% |
|  | 20 | 24.9 | 2.05% | 10.334 | 0.54% |

Supplementary Table S2 Analytical sensitivity of MAGLUMI HBeAg (CLIA) and MAGLUMI Anti-HBe (CLIA) assays

| Sample ID | Average  (IU/mL) | interpretation | Assigned value IS PEI code 129097/12(IU/mL) | Sample ID | average  (AU/mL) | interpretation | Assigned value IS PEI code 129095  (IU/mL) |
| --- | --- | --- | --- | --- | --- | --- | --- |
| HBeAgd1 | 1.004 | Reactive | 1 | Anti-HBed1 | 12.12 | Reactive | 2 |
| HBeAgd2 | 0.526 | Reactive | 0.5 | Anti-HBed2 | 6.65 | Reactive | 1 |
| HBeAgd3 | 0.270 | Reactive | 0.25 | Anti-HBed3 | 2.77 | Reactive | 0.5 |
| HBeAgd4 | 0.149 | Reactive | 0.125 | Anti-HBed4 | 1.19 | Reactive | 0.25 |
| HBeAgd5 | 0.076 | Non-Reactive | 0.0625 | Anti-HBed5 | 0.43 | Non-Reactive | 0.125 |
| HBeAgd6 | 0.041 | Non-Reactive | 0.03125 | Anti-HBed6 | 0.20 | Non-Reactive | 0.0625 |
| HBeAgd7 | 0.023 | Non-Reactive | 0.0156 | Anti-HBed7 | 0.06 | Non-Reactive | 0.03125 |

Supplementary Table S3 Detailed results of initial false-positive samples on the MAGLUMI Anti-HBe (CLIA)

| Sample ID | MAGLUMI Anti-HBe (AU/mL) | | | Architect Anti-HBe (S/CO) | Cobas  Anti-HBe (COI) |
| --- | --- | --- | --- | --- | --- |
|  | Initial | Repeat 1 | Repeat 2 |  |  |
| 1512940032 | 1.201 (reactive) | 1.593  (reactive) | 1.556  (reactive) | 2.19  (non-reactive) | 2.09  (non-reactive) |
| 7976370383 | 1.015  (reactive) | 1.099  (reactive) | 1.046  (reactive) | 2.04  (non-reactive) | 1.89  (non-reactive) |

Supplementary Table S4 Detailed results of false-negative samples on the MAGLUMI Anti-HBe (CLIA)

| Sample ID | MAGLUMI Anti-HBe (AU/mL) | | | Architect  Anti-HBe  (S/CO) | Cobas  Anti-HBe  (COI) |
| --- | --- | --- | --- | --- | --- |
|  | Initial | R1 | R2 |  |  |
| 17259 | 0.498  (non-reactive) | 0.768  (non-reactive) | 0.808  (non-reactive) | 0.81  (reactive) | 0.69  (reactive) |
| 19032263 | 0.501  (non-reactive) | 0.524  (non-reactive) | 0.475  (non-reactive) | 0.87  (reactive) | 0.70  (reactive) |
| K01088-E | 0.943  (non-reactive) | 0.981  (non-reactive) | 0.897  (non-reactive) | 0.77  (reactive) | 0.73  (reactive) |
| K10061-E | 0.853  (non-reactive) | 0.970  (non-reactive) | 0.992  (non-reactive) | 0.73  (reactive) | 0.83  (reactive) |
| K10377-E | 0.952  (non-reactive) | 1.025  (non-reactive) | 0.957  (non-reactive) | 0.84  (reactive) | 0.64  (reactive) |

Supplementary Table S5 Negative Serum-plasma equivalency of MAGLUMI HBeAg (CLIA) and MAGLUMI Anti-HBe (CLIA) assays

| Sample type |  | MAGLUMI HBeAg(CLIA) | | MAGLUMI Anti-HBe(CLIA) | |
| --- | --- | --- | --- | --- | --- |
|  |  | Reactive | Non-reactive | Reactive | Non-reactive |
| Serum |  | 0 | 25 | 0 | 25 |
| Plasma | Sodium-Citrate | 0 | 25 | 0 | 25 |
|  | K2EDTA | 0 | 25 | 0 | 25 |
|  | K3EDTA | 0 | 25 | 0 | 25 |
|  | Lithium Heparin | 0 | 25 | 0 | 25 |
|  | Sodium Heparin | 0 | 25 | 0 | 25 |
|  | ACD-B | 0 | 25 | 0 | 25 |
|  | CPDA | 0 | 25 | 0 | 25 |
|  | K-Oxalate/NaF | 0 | 25 | 0 | 25 |
|  | CPD | 0 | 25 | 0 | 25 |

Supplementary Table S6 Passing-Bablok regression results between measurements in serum and different plasma types for the MAGLUMI HBeAg (CLIA)

| Plasma | Correlation coefficient | Slope | 95% CI of  slope | Intercept | 95% CI of Intercept |
| --- | --- | --- | --- | --- | --- |
| ACD-B | 0.987 | 1.073 | 1.005-1.106 | -0.008 | -0.320-0.030 |
| CPD  (without outliers) | 0.995 | 0.988 | 0.947-1.052 | 0.027 | -0.024-0.141 |
| CPD  (with outliers) | 0.989 | 0.987 | 0.947-1.051 | 0.019 | -0.027-0.106 |
| CPDA | 0.987 | 1.065 | 0.948-1.112 | -0.011 | -0.053-0.097 |
| K2EDTA | 0.996 | 1.058 | 0.987-1.126 | -0.015 | -0.105-0.045 |
| K3EDTA | 0.990 | 1.069 | 1.003-1.151 | -0.006 | -0.055-0.061 |
| Li Heparin | 0.993 | 1.051 | 1.021-1.126 | -0.036 | -0.137-0.007 |
| Sodium citrate (without outliers) | 0.985 | 1.042 | 0.924-1.100 | -0.057 | -0.138-0.030 |
| Sodium citrate (with outliers) | 0.975 | 1.041 | 0.894-1.100 | -0.058 | -0.213-0.046 |
| K-Oxalate/NaF | 0.986 | 1.024 | 0.923-1.136 | 0.022 | -0.042-0.080 |
| Sodium Heparin | 0.994 | 1.055 | 0.993-1.117 | -0.049 | -0.201-0.010 |

Supplementary Table S7 Whole system failure rate of MAGLUMI HBeAg (CLIA) and MAGLUMI Anti-HBe (CLIA) assays

| assay | Sample | False negative | Reactive | Total tests |
| --- | --- | --- | --- | --- |
| MAGLUMI HBeAg assay | sample 1 | 0 | 40 | 40 |
|  | sample 2 | 0 | 40 | 40 |
|  | sample 3 | 0 | 40 | 40 |
| MAGLUMI  Anti-HBe assay | sample 1 | 0 | 40 | 40 |
|  | sample 1 | 0 | 40 | 40 |
|  | sample 3 | 0 | 40 | 40 |

Supplementary Figure S1 Linear range of MAGLUMI HBeAg (CLIA)

Supplementary Figure S2 Passing-Bablok regression analysis for positive Serum-plasma equivalency of MAGLUMI HBeAg (CLIA)
